# Benefits and Challenges of Telerehabilitation Use By Pediatric Physiotherapists During the COVID-19 Pandemic In Western and Southern India: A Cross-Sectional Survey

**DOI:** 10.5195/ijt.2022.6466

**Published:** 2022-06-03

**Authors:** Shukra Chivate, Mehek Sharma, Aamila Shaikh, Chandrika Satarkar

**Affiliations:** Kaher Institute Of Physiotherapy, Belagavi, Karnataka, India

**Keywords:** COVID-19, Pediatric, Physiotherapists, Telehealth, Telerehabilitation

## Abstract

As the worldwide COVID-19 pandemic spread, many physiotherapists chose telerehabilitation (TR) to continue delivering therapy. This study was conducted to document the perceived benefits and challenges of TR faced by pediatric physiotherapists in western and southern India. Using the snowball method, electronic survey forms were distributed to 275 pediatric physiotherapists in Western and Southern India; 110 responses were available for analysis. A majority of respondents had experience with TR (n=83, 75.5%), while others had never used TR (n=27, 24.5%). TR was reported to be less effective than in-person therapy for treating children and adolescents during the COVID-19 pandemic. Therapists reported significant difficulties during TR sessions; these included assessing and modifying exercises for children. As the popularity of TR grows, pediatric physiotherapists will need to be aware of the benefits and challenges they will face during TR sessions. Most pediatric physiotherapists believed the interaction between parents and therapists is a requisite for optimal service delivery.

COVID-19 emerged as a cluster of respiratory infections in Wuhan, China, and soon spread worldwide, prompting the United States (U.S.) to declare a state of emergency on March 13, 2020. By April 4, 2020, the World Health Organization reported more than one million cases of the virus worldwide (Hall et al., 2021). To restrict the spread of the virus, governments and authorities in affected nations implemented preventive measures such as enforced lockdowns, social distancing, self-isolation, and quarantine (Shah et al., 2020).

As the global pandemic progressed, the U.S. Centers for Disease Control and Prevention urged that health care practitioners use procedures such as telehealth to decrease contact and the danger of virus spread (Hall et al., 2021). Similarly, the board of directors of the American Physical Therapy Association (APTA), urged physiotherapists to “use their professional judgement to select when, where, and how to administer care, for anyone involved” (Hall et al., 2021, p. 237). Many physiotherapists chose telerehabilitation (TR) to continue giving care while reducing in-person interactions to keep the virus from spreading (Hall et al., 2021). Hence, the rapid adoption of TR was vital for pediatric developmental therapy to continue to provide safe and high-quality treatment services to vulnerable populations (Hall et al., 2021)

Although physiotherapy has been suggested for patients with COVID-19 in both acute and long-term settings, there have been challenges to recommendations to implement TR in other areas where physiotherapists work. Pediatric physiotherapy is one such service that has been severely impacted. Children were confined to their homes as playgrounds were closed and schools became virtual. Changes in their lifestyle, combined with the psychological stress of being confined at home, may negatively impact children with impairments. As a result, there was a need to provide other delivery options for physiotherapy treatments to children with disabilities during this time of limited access to rehabilitation services (Rao, 2021).

Rehabilitation is required to enhance people's functioning and quality of life by improving their abilities to live, work, and learn. The consequences of disability can negatively affect a neighborhood, society, and the economy. When movement and function are endangered, physiotherapy interventions are needed to build, preserve, and re-establish movement and functional capacity, with the understanding that functional movement is vital to health and an optimal quality of life (Seron et al., 2021).

Because of a paradigm shift in illness load during COVID-19, India experienced enormous problems in the health care sector (Khanna et al., 2018). The release of Telemedicine Guidelines by The Medical Council of India (MCI) on March 25, 2020, and the ongoing COVID-19 pandemic, resulted in growth in telemedicine services across the country, with doctors becoming more confident in delivering such services. To keep the virus from spreading, it was best to use telemedicine whenever possible (Kumari et al., 2021).

The research on telehealth was slowly growing as technology advanced prior to the COVID-19 pandemic. It has now become a critical approach for providing healthcare while minimizing the spread of the virus (Hall et al., 2021). Specialized fields of telehealth include e-health, telemedicine, telematics, and TR (Movahedazarhouligh et al., 2015). TR, a branch of telehealth, is defined as using information and communication technologies (ICT) to provide rehabilitation services to people remotely in their homes or other environments (Brennan et al., 2015). TR can be delivered by a number of methods, including two-way real-time audio visits, video visits, or both; asynchronous e-visits, virtual check-ins, remote assessments of recorded films or photos, and telephone assessment and management services (Bettger et al., 2020). TR therapies are less expensive, take less time, and are often more convenient (Egmond et al., 2018).

TR services have been used to treat conditions such as stroke, traumatic brain injury, multiple sclerosis, chronic pain, hip and knee total arthroplasty, and medical conditions such as congestive heart failure and rheumatologic disease, both by itself and in conjunction with conventional care (Dietzen et al., 2020).

TR visits relieve the strain of taking the child's medical equipment to a routine check-up, arranging for other children to be cared for at home, and taking time off work for families of disabled children. Virtual visits offer a unique opportunity to understand the obstacles and constraints that patients face at home, and the potential for more targeted solutions (Rabatin et al., 2020). Given the possibility of TR to enhance rehabilitation potential and the paucity of studies on TR in the pediatric population in the literature, more information on this topic is needed (Krasovsky et al., 2021).

Virtual visits may become part of our ongoing paradigm of care and may impact the frequency of in-person visits if the constraints in delivering TR and telemedicine are permanently removed (Rabatin et al., 2020). As of this date, there has been a lack of research on the deployment and distribution of TR services among children with physical and cognitive impairments (Krasovsky et al., 2021). Because of the increased use of TR, it is critical to understand the benefits and problems that pediatric physiotherapists encounter during online treatment sessions.

This study aimed to determine the benefits and challenges faced by pediatric physiotherapists, to learn of the perceived effectiveness of their TR sessions and enhance future sessions.

## SUBJECTS AND METHODS

Ethical clearance was obtained from the institutional ethical committee prior to the commencement of the study. Pediatric physiotherapists in Western and Southern India participated in the study. Various pediatric physiotherapy institutes, clinics, and hospital units throughout Western and Southern India were contacted through emails and social media. A list was made based on the inclusion and exclusion criteria, and by using the snowball method, more pediatric physiotherapists were contacted. The snowball method involves a participant identifying other potential participants who have knowledge of the topic under study. An electronic survey form was sent to practicing pediatric physiotherapists in Western and Southern India. A set of questions was displayed when a pediatric physiotherapist chose “YES” to indicate prior TR experience. In contrast, those without prior TR experience who chose the response “NO”, indicating no experience with TR, had one multiple choice question to answer on the survey. The form consisted of four sections. Section A contained a consent form to participate in the study. Section B contained seven basic questions pertaining to demographic variables (i.e., name, email address, identification number, state, years of practicing pediatric physiotherapy, specialization, and TR experience). Section C consisted of 15 questions for those who had experienced TR. Section 4 consisted of one multiple choice question for those with no TR experience. Out of 275 forms sent, 110 responses were received. The survey was conducted over a 3-month period between December 2021 – February 2022.

## RESULTS

Table 1 outlines demographic data based on number of responses received from different states.

**Table 1:** Number of Responses Received from Different States

States	Responses	Percentage
MAHARASHTRA	47	42.72%
KARNATAKA	21	19.09%
GUJARAT	18	16.66%
GOA	5	4.62%
TAMIL NADU	5	4.62%
ANDHRA PRADESH	4	3.70%
RAJASTHAN	4	3.70%
TELANGANA	3	2.77%
KERALA	3	2.77%
TOTAL	110	100%

Table 2 outlines the responses received for participants with TR experience for each survey question and includes response frequency and percentage.

**Table 2:** Descriptive Data of Survey Question Responses for Participants with TR Experience

SR. NO	Statement	Responses	Frequency (N)	Percentage (%)
1	Parent-therapist interaction is critical for optimal service delivery in pediatric rehabilitation.	Strongly agree	59	76.6
		Agree	12	15.6
		Neutral	3	3.9
		Disagree	2	2.6
		Strongly disagree	1	1.3
2	Hands-on approach is required for efficient service delivery	Strongly agree	52	67.5
		Agree	19	24.7
		Neutral	3	3.9
		Disagree	3	3.9
		Strongly disagree	-	-
3	The use of therapeutic equipment is essential in the treatment of children	Strongly agree	19	24.7
		Agree	31	40.3
		Neutral	19	24.7
		Disagree	6	7.8
		Strongly disagree	2	2.6
4	Home setting is appropriate for pediatric treatment sessions	Strongly agree	7	9.1
		Agree	19	24.7
		Neutral	36	46.8
		Disagree	15	19.5
		Strongly disagree	-	-
5	Telerehabilitation is a convenient mode to deliver therapy sessions	Strongly agree	1	1.3
		Agree	14	18.2
		Neutral	31	40.3
		Disagree	28	36.4
		Strongly disagree	3	3.9
6	Telerehabilitation saves travel time for both patient as well as therapist	Strongly agree	10	13
		Agree	38	49.4
		Neutral	24	31.2
		Disagree	4	5.2
		Strongly disagree	1	1.3
7	Technical difficulties faced during therapy sessions has hampered delivery of services	Strongly agree	25	
		Agree	36	32.5
		Neutral	11	46.8
		Disagree	5	14.3
		Strongly disagree		6.5
8	Telerehabilitation sessions require a stable internet connection and access to necessary gadgets.	Strongly agree	49	63.6
		Agree	27	35.1
		Neutral	-	-
		Disagree	1	1.3
		Strongly disagree	-	-
9	It is necessary to provide parents with prior training to telerehabilitation sessions	Strongly agree	38	49.4
		Agree	32	41.6
		Neutral	4	5.2
		Disagree	3	3.9
		Strongly disagree	-	-
10	Therapy sessions via telerehabilitation lower the quality of treatment and care	Strongly agree	19	24.7
		Agree	30	39
		Neutral	20	26
		Disagree	7	9.1
		Strongly disagree	1	1.3
11	It is mentally exhausting for therapists to conduct telerehabilitation sessions	Strongly agree	16	20.8
		Agree	27	35.1
		Neutral	18	23.4
		Disagree	16	20.8
		Strongly disagree	-	-
12	It is challenging to give instructions to children via telerehabilitation sessions.	Strongly agree	29	37.7
		Agree	37	48.1
		Neutral	10	13
		Disagree	1	1.3
		Strongly disagree	-	-
13	It is important to conduct therapy sessions in a distraction-free atmosphere via telerehabilitation	Strongly agree	33	42.9
		Agree	38	49.4
		Neutral	5	6.5
		Disagree	-	-
		Strongly disagree	1	1.3
14	Telerehabilitation makes it difficult to access and modify exercise for children	Strongly agree	21	27.3
		Agree	35	45.5
		Neutral	9	11.7
		Disagree	12	15.6
		Strongly disagree	-	-
15	Telerehabilitation is a successful method for providing therapy to children.	Strongly agree	1	1.3
		Agree	19	24.7
		Neutral	30	39
		Disagree	21	27.3
		Strongly disagree	6	7.8

Of the physiotherapists that participated in this survey across Western and Southern India, Maharashtra garnered the most responses, with 47 (42.72%), followed by Karnataka with 21 (19.09%), Gujarat with 18 (16.66%), Goa and Tamil Nadu each with 5 (4.62%), Andhra Pradesh and Rajasthan each with 4 (3.70%), and Telangana and Kerala each with 3 (2.77%) responses.

Among the 110 respondents, 83 (75.5%) had prior experience with TR, whereas 27 (24.5%) had never used TR.

A majority of pediatric physiotherapists, 71 (85.5%), agreed with the statement that a good connection between parents and therapists is crucial for optimal service delivery in pediatric physiotherapy, 71 (85.5%) agreed that hands-on approach is required for efficient service delivery and 50 (%) agreed to the statement that the use of therapeutic equipment (e.g., elastic bands, medical balls, weights) is essential in the treatment of children. A total of 48 (57.8 %) people agreed that TR saves travel time for patients as well as the therapist and 76 (91.5%) participants agreed that TR requires a stable internet connection and necessary gadgets such as a computer with camera and microphone, laptop, mobiles etc.

Table 3 outlines the responses received for participants without TR experience for each survey question and includes response frequency and percentage.

**Table 3:** Descriptive Data of Survey Question Responses for Participants without TR Experience

SR. NO	Question	Responses	Frequency (N)	Percentage (%)
1	Why have you not considered telerehabilitation as a mode of practice?	1. Technical difficulties faced during TR will hamper delivery of therapy.	8	24.2
		2. Therapy via TR reduces quality of treatment and care.	19	57.6
		3. It is difficult to assess and modify exercises for children during TR sessions.	21	63.6
		4. It is challenging to give instructions to children during TR.	16	48.5
		5. TR will make it difficult for therapists and parents to communicate.	6	18.2
		6. It causes difficulty in effective delivery of consultation and therapy.	10	30.3
		7. Poor internet connectivity is the biggest issue in rural part of India which hampers sessions.	1	3

The following challenges were indicated for therapy using TR: difficulty assessing and modifying exercises for children during sessions (63.6%) and the perception of reduced quality of treatment and care (57.6%).

## DISCUSSION

This study was carried out to discover the benefits and drawbacks of TR among pediatric physiotherapists in Western and Southern parts of India during the pandemic. The majority of the 110 participants indicated having experience with TR. Parent-therapist interaction was found to be critical for optimal service delivery.

Reported challenges of TR included technological difficulties, unstable internet connections, and difficulty in delivering instructions to children via TR.

TR use can bring significant benefits, such as greater access to services, improved access to specialized clinicians or experts, and the avoidance of needless delays in getting therapy. During the initial weeks and months of the COVID-19 pandemic, TR provided a safe, accessible style of therapy that reduced travel to hospital settings while encouraging social distancing among outpatients and staff. Rapid TR adoption enabled therapists to continue providing therapy while retaining patient satisfaction (Tanner et al., 2020)

A substantial amount of research is available that discusses the use of TR in various sectors of physiotherapy. One such research study of 176 respondents conducted by D’Souza et al. (2021) concluded that TR is a practical strategy because it saves time and money while also easing the pressure on physiotherapists. Most of their respondents said that they were satisfied and confident using information and communication technology (ICT). They also faced a number of challenges, including lack of training, lack of connections between ICT experts and practitioners, worries about the cost of data protection and equipment, and a lack of user-friendly software (D'Souza et al., 2021). A similar study conducted in Kuwait found that the absence of suitable technology, poor network coverage, and lack of hospital and IT assistance were the main impediments to the successful use of TR. Most respondents felt that they could generally trust technology to work and were content to use ICT. Most respondents stated that they are willing to use TR to deliver physiotherapy (Albahrouh et al., 2021).

The findings of our research are consistent with the aforementioned studies in that TR saved travel time for both the therapist and patient. Similar challenges were also seen in our study, such as lack of training for the parents prior to treatment sessions and unstable internet connection. We cannot remark on the viability of TR because a large proportion of respondents in our study were neutral about its convenience and success. Hence, a larger sample size would be required to comment on that. One of the primary reasons some of the physiotherapists in our study did not use TR was because they thought it harmed the quality of therapy and care they provided. This could be due to the difficulty in giving instructions to children via TR, technical difficulties, and unreliable internet connections.

In contrast, a prior study conducted in Saudi Arabia stated that the implementation of TR in the physiotherapy setting would improve the quality of healthcare. However, technical challenges, staff skill issues, high cost, shortage of experienced personnel, and patient compliance were the main impediments to TR deployment in that study (Aloyuni et al., 2020).

A study by Turolla et al. (2020) of musculoskeletal physiotherapists observed that TR benefits included maintaining continuity of care, educating patients via virtual consultation, monitoring patients' improvement, and increasing care efficiency while controlling costs and minimizing waiting lists, all of which helped to ensure that services were sustainable. At the same time, a difficulty they encountered was the inability to use palpation or other specialized tests as diagnostic tools; this jeopardized the screening procedure. As a result, an in-person first-contact visit should be carefully considered for patients who require a higher level of care. Another challenge they faced was the lack of rehabilitation equipment (e.g., elastic bands, medical balls, weights) to deliver musculoskeletal care. Hence, they concluded that physiotherapists should determine which tools are required for therapeutic activity at the initial planning stages of the rehabilitation program and then rent and transport them to the patient's home (Turolla et al., 2020). In our research, we noticed a similar emphasis on hands-on approach and therapeutic tools for providing care efficiently.

TR increased intervention time for patients with neurological problems, according to Suso-Martí et al. (2021) because travel time was minimized. TR also allowed patients to train functional tasks in their environment rather than in a clinical setting, therefore facilitating the transfer of skills to regular life; this is critical for patients with neurological disorders. Our current study determined that home treatment sessions were appropriate for pediatric treatment sessions since the child was in a familiar environment. Furthermore, TR was found to reduce both patient and therapist travel time.

Pediatric physiotherapists throughout the U.S. stressed the need for caregiver involvement in a successful telehealth session (Hall et al., 2021). This requires the caregiver to be an active participant in all facets of the therapy sessions, and not just have a presence in the room. The level of parental engagement was influenced by the personalities of the caregiver and the clinician, their communication styles, and the quality of their interactions. This engagement was enhanced by a parent coaching model, which some indicated as an essential factor in encouraging caregiver success during TR sessions. “Parent coaching” is defined as a collaborative effort in which a physiotherapist assists a caregiver in making decisions to strengthen their method of engagement. Parent coaching was found to be successful for pediatric physiotherapy, especially for early intervention. Yet, challenges persisted. Parents complained of “Zoom fatigue” when several children in the family were engaged in online schooling. The stress of caregivers ill with COVID-19 was also mentioned by respondents (Hall et al., 2021). The physiotherapists' TR sessions for the pediatric population were found to be mentally tiring in our study. Perhaps the difficulty of giving instructions to children at a distance could be one of the causes.

The benefits and limits of existing physiotherapy digital practice were outlined by Hall et al. (2021). Benefits included more service accessibility, no travel time, increased schedule flexibility, end-user control over healthcare service, and lower cost. Challenges cited related to internet and device access, environmental setup, cultural issues, communication obstacles, training, finance, and regulatory factors.

According to Hall et al. (2021) the majority of the responders said that offering telehealth in the midst of the pandemic had been a beneficial experience for them. The essential factors for telehealth efficacy were high caregiver participation and access to dependable technology. The respondents recommended that pediatric physiotherapists be trained in the use of family-centered coaching, as well as how to achieve universal internet and device access. They recommended that health care and government agencies collaborate with insurance companies to facilitate telehealth use (Hall et al., 2021). If these same facilitators were to be employed in India, TR may become an even greater viable alternative to in-person therapy.

Hall et al. (2021) found that TR effectiveness and willingness of therapists to use TR were lower for school-based physiotherapists than for physiotherapists in outpatient and early intervention settings. Telehealth may be easily deployed in all of these situations, and may bring benefits like increased flexibility, physician access, and caregiver engagement (Hall et al., 2021). In the current study for pediatric physiotherapists, only a small percentage believed TR is a practical and effective service delivery model, with the majority remaining undecided. Overall, our research found that a minority (39.7%) of pediatric physiotherapists agreed that TR is a convenient and successful technique for delivering physical therapy to children. A study spanning all Indian states is needed.
